# Respiratory virus associated with surgery in children patients

**DOI:** 10.1186/s12931-019-1086-y

**Published:** 2019-06-17

**Authors:** Dan Zhang, Xiuyu Lou, Hao Yan, Junhang Pan, Haiyan Mao, Hongfeng Tang, Yan Shu, Yun Zhao, Lei Liu, Junping Li, Dong Chen, Yanjun Zhang, Xuejun Ma

**Affiliations:** 1 NHC Key Laboratory of Medical Virology and Viral Diseases, National Institute for Viral Disease Control and Prevention, Chinese Center for Disease Control and Prevention, No.155 Changbai Road, Changping district, Beijing, 102206 China; 2 Institute of Microbiology, Zhejiang Provincial Center for Disease Control and Prevention, No. 3399 Binsheng Road, 310051, Binjiang district, Hangzhou, China; 3 Department of Pathology, The Children’s Hospital, Zhejiang University School of Medicine, Hangzhou, China; 4 Department of Laboratory Medicine, The Sixth People Hospital of Wenzhou, Wenzhou, China

**Keywords:** Respiratory virus, Pediatric surgical patients, Symptoms, mRT-PCR

## Abstract

**Background:**

Viral respiratory infection (VRI) is a common contraindication to elective surgery. Asymptomatic shedding among pediatric surgery patients (PSPs) could potentially lead to progression of symptomatic diseases and cause outbreaks of respiratory diseases. The aim of this study is to investigate the incidence of infection among mild symptomatic PSP group and asymptomatic PSP group after surgical procedure.

**Methods:**

We collected the induced sputum from enrolled 1629 children (under 18 years of age) with no respiratory symptom prior to pediatric surgery between March 2017 and February 2019. We tested 16 different respiratory virus infections in post-surgery mild symptomatic PSP group and asymptomatic PSP group using a quantitative real-time reverse transcriptase polymerase chain reaction (qRT-PCR) assay panel. We analyzed symptom data and quantitative viral load to investigate the association between viruses, symptoms and viral quantity in qRT-PCR-positive PSPs.

**Results:**

Out of 1629 children enrolled, a total of 204 respiratory viruses were present in 171 (10.50%) PSPs including 47 patients with mild symptoms and 124 with no symptoms after surgery. Commonly detected viruses were human rhino/enterovirus (HRV/EV, 42.19%), parainfluenza virus 3 (PIV3, 24.48%), coronavirus (CoV NL63, OC43, HKU1, 11.46%), and respiratory syncytial virus (RSV, 9.9%). PIV3 infection with a higher viral load was frequently found in PSPs presenting with mild symptoms, progressing to pneumonia with radiographic evidence after surgery. HRV/EV were the most commonly detected pathogens in both asymptomatic and mild symptomatic PSPs. CoV (OC43, HKU1) infections with a higher viral load were mostly observed in asymptomatic PSPs progressing to alveolar or interstitial infiltration.

**Conclusions:**

Our study suggested that PIV3 is a new risk factor for VRI in PSPs. Employing a more comprehensive, sensitive and quantitative method should be considered for preoperative testing of respiratory viruses in order to guide optimal surgical timing.

## Background

Respiratory virus is well known to cause significant morbidity and mortality in pediatric populations [1–3]; especially in neonates or very young infants; in patients with chronic heart or lung disease; and in the immunosuppressed [4]. Numerous studies have described viral respiratory infection (VRI) as a leading cause of illness and death in people of all ages [5]. The most frequent community-acquired respiratory viruses are respiratory syncytial virus (RSV), parainfluenza virus (PIV), human influenza virus (IV), human metapneumovirus (HMPV), and human rhinovirus (HRV) in the respiratory infection patients, hematopoietic cell transplant recipients, and hematologic malignancy patients with an immunocompromised status [3, 6–9].

Most observations of human respiratory virus carriage are derived from medical surveillance. As VRIs can often be mild or asymptomatic and typically go undocumented, individuals may not seek medical care because either the infection is mild or it elicits symptoms not severe enough to warrant contacting a medical professional [10]. Asymptomatic viral shedding in the nasopharynx in asymptomatic populations and immunocompetent hosts is relatively common [11–13]. Asymptomatic shedding among pediatric surgery patients (PSPs) could potentially play an key role in propagating outbreaks of respiratory diseases if subclinical VRI is transmissible and leads to progression of symptomatic disease [13]. However, the infections reported by the surveillance showed only the symptomatic fraction of the total infected population [14]. The role of asymptomatic infection in respiratory virus transmission is still largely unknown and the rates of asymptomatic shedding are not well studied.

The issue of preoperative VRI screening among children undergoing surgery is unclear and complex. If children with VRI presented with obvious symptoms (fever, cough), it would be an easy decision for the surgeon or anesthesiologist to delay surgery if appropriate. In cases of children with mild or no symptoms, it becomes very difficult for doctors to make the right decision. A few studies have investigated VRI associated with a limited number of respiratory viruses and have demonstrated an increased length of stay (LOS) and increased postoperative complication rates in pediatric cardiac surgical patients [15, 16]. The data for VRI among the PSPs is not performed systematically recorded and thus detailed etiologic data are lacking. To better estimate the current state of VRI in PSPs and to improve clinical management, this study aims to investigate the incidence of VRI in the mild symptomatic and asymptomatic pediatric surgery population after surgical procedures and to evaluate the impact of VRI on postoperative outcomes. We analyzed detailed symptom data using a standardized symptom survey and quantitative viral load using multiple qRT-PCR to determine the associations between viruses, symptoms and viral quantity.

## Methods

### Study design and patients

This was a retrospective surveillance study of pediatric patients after surgery at the Children’s Hospital, Zhejiang University School of Medicine, China. Children (age < 18 year) undergoing a surgical procedure between March 2017 and February 2019 were eligible for enrollment. We obtained induced sputum specimens from hospitalized patients within 48 h of admission regardless of the presence of respiratory symptoms, as part of standard care in the hospital. If pediatric patients had serious symptoms suggestive of respiratory infections before surgery and must delay surgery, these patients were excluded from analysis. The PSPs who had no respiratory symptom prior to elective or emergency surgery were included in the study. After surgery, study subjects completed a baseline information form and a standardized symptom survey that included six symptoms (fever [17], wheezing, sputum, shortness of breath, soar throat and cough) and radiographic findings (alveolar or interstitial infiltrate and pleural effusion). Fever was defined as a body temperature ≥ 38.0 °C. Mild symptoms refer to the subjects who had at least one of the above respiratory symptoms that were not severe. A clinical chart review was performed for subjects to determine whether symptoms occurred outside of the study visit. Data on management and outcome of the cases were collected from the medical records. The study was approved by the institutional ethics review board at the Center for Disease Control and Prevention of Zhejiang, including a waiver of informed consent.

### Virologic methods

Sputum samples were collected from each patient in the hospital. The samples were subsequently divided into two aliquots and stored at − 80 °C until required for the assay. Nucleic acids were extracted from 200-µL samples using the cador Pathogen 96 QIAcube HT Kit (Qiagen) for automated viral DNA and RNA extraction with the QIAcube HT System. The samples were tested individually by qRT-PCR using specific primers and probes targeting different genomes according to our previously reported qRT-PCR methods [18]. The qRT-PCR assays were used to detect 16 different respiratory viruses: CoV (229E, NL63, OC43 and HKU1), PIV1–4, IV types A and B, RSV types A and B, HRV/EV, HMPV, human adenovirus (ADV), and human bocavirus (HBoV).

### Comparison of Ct values

qRT-PCR threshold cycle (Ct) represents the first PCR cycle in which fluorescent signal for the target (eg: viral RNA) is greater than the minimal detection level. Ct values are inversely related to the viral load, thus offering a semiquantitative assessment. Lower Ct values were considered to reflect a higher viral load than the higher Ct values. We compared Ct values between mild symptomatic and asymptomatic PSPs for different viruses in each positive sample when viruses were detected in PSPs with a prevalence of > 5%. Statistical analyses were performed using Stata 13 software. Differences were considered significant when *p* < 0.05 (two-sided).

### Statistical methods

We summarized and compared surgical patients demographic data between respiratory virus-positive and virus-negative groups using chi-square or Fisher’s exact test for categorical variables and Wilcoxon rank-sum test for continuous variables, as appropriate. We used Student-Newman-Keuls test to assess whether there were statistical differences in the Ct values of different respiratory viruses between mild symptomatic and asymptomatic PSPs. For categorical variables, such as age group and gender, we used Fisher’s exact test, ANOVA and *Mann-Whitney U test* to compare the differences across each categorical variable with respect to positivity. Each virus detected was counted individually. For example, in the case of co-detection of HRV and PIV3, detection of each virus was considered positive in separate HRV and PIV3 prevalence calculations.

## Results

### Respiratory virus incidence

**Fig. 1:**
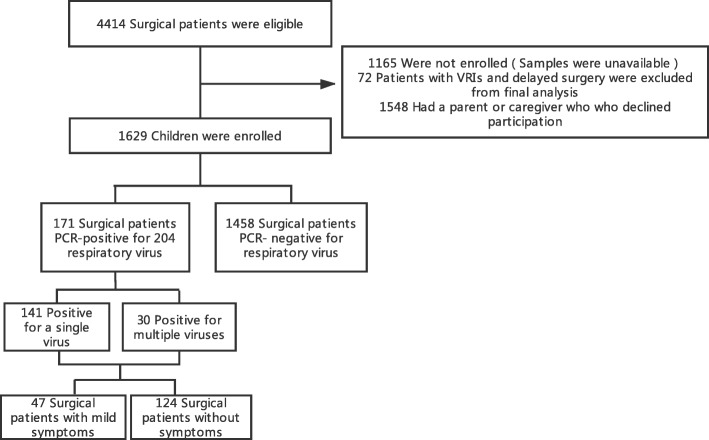
Consort flow diagram. (Eligibility and Enrollment of Surgery Patients)

Of the 4414 eligible children, 1629 patients were enrolled. We surveyed 1629 patients undergoing a surgical procedure during the study period (Fig. 1). Among them, 52.87% of participants were female and 47.13% were male. Respiratory samples from 171 (10.50%) of 1629 subjects had one or more respiratory viruses detected (Table 1), of which, 47 (2.88%) subject were from mild symptomatic group and 124 (7.62%) from asymptomatic group (Fig. 1). There was no difference in median age between qRT-PCR-positive and -negative patients. The epidemiology of the respiratory disease varied substantially among the different pediatric age group (Table 1). The median LOS of qRT-PCR-positive and -negative patients in the entire cohort was 18.0 days (IQR, 11.0–27.0) and 13.0 days (IQR, 9.0–21.0), respectively. Thus, patients with viral respiratory illness were more likely to have a longer postoperative LOS (*p* < 0.001, Table 1).

**Table 1:** Comparison of baseline demographic data and surgical procedural data according to the qRT-PCR results

Patient demographics	qRT-PCR Positive (*n* = 171)	qRT-PCR Negative (*n* = 1458)	*p*
Age (mo), median (IQR)	8(3.6–22.4)	9.6(3.0–30.6)	0.562
Age category, n. (%)			< 0.001
Neonates age < 29 d	0(0)	98(6.72)	
29d–3 yr	88(51.46)	919(63.03)	
> 3 yr	83(48.54)	441(30.25)	
Male, n. (%)	82(48.04)	797(54.67)	0.076
Length of stay, median (IQR)	18.0(11.0–21.0)	13.0(9.0–21.0)	< 0.001
Death in the hospital, no. (%)	4(< 1)	3(< 1)	0.006

IQR = interquartile range. Continuous variables are represented as median and IQR, and categorical variables are summarized as *n* (%)

**Fig. 2:**
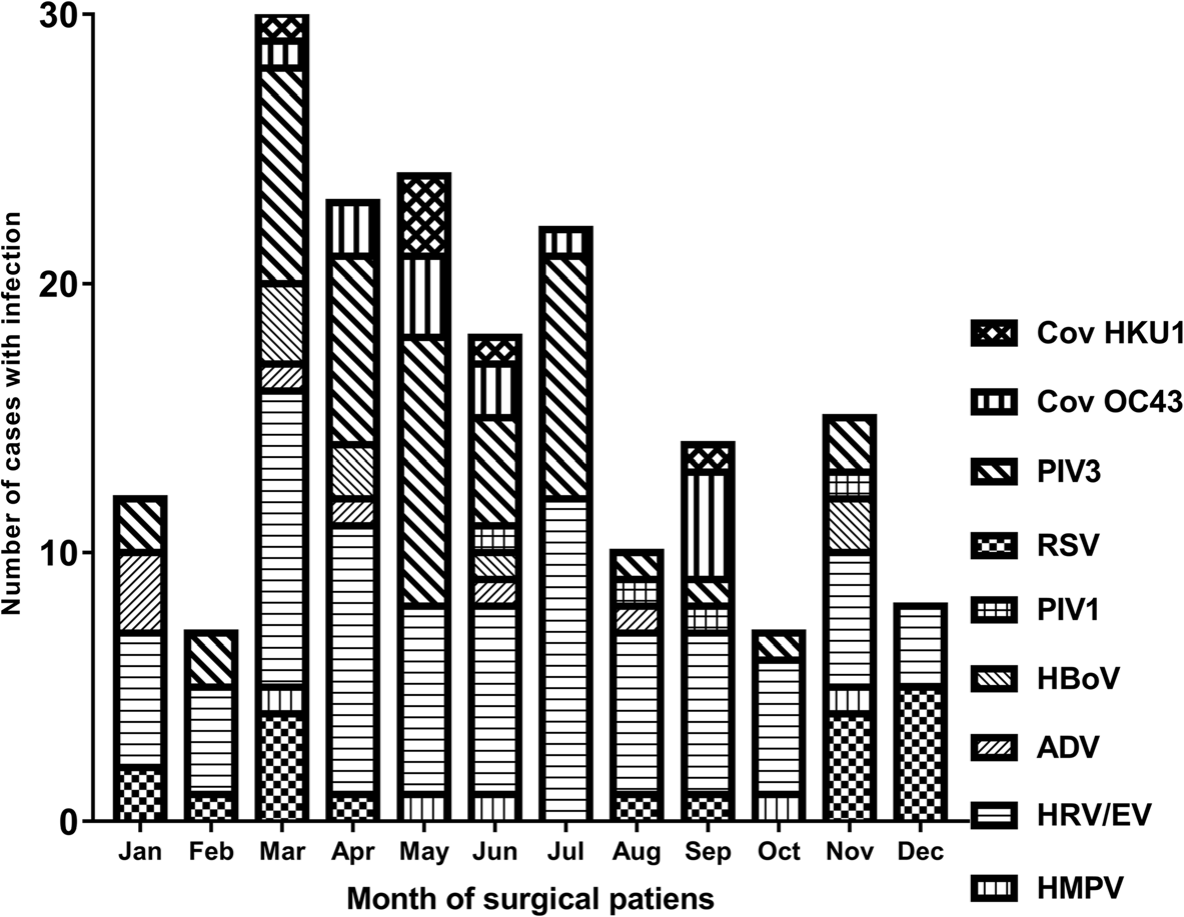
Histogram showing the frequency and type of pathogen (*left axis*) according to month, in Zhejiang pediatric patients in the period from March 2017 through February 2019

A total of 204 respiratory viruses were detected in 171 PSPs. A single virus was detected in 141 PSPs (8.66%), and 30 PSPs (1.84%) had multiple viruses identified, which was involved with 63 respiratory viruses. The most commonly identified viruses were HRV/EV (81,42.19%), followed by PIV3 (47, 24.48%), RSV (19, 9.90%), CoV OC43 (13, 6.77%), IV (10, 5.2%), HboV (8, 4.17%), and CoV HKU1 (8, 4.17%) (Table 2). HRV/EV showed the highest rate of infection among respiratory viruses and accounted for 25(12.25%) detected co-infections. The most commonly detected co-infections were HRV/EV and PIV3 (15,7.35%). CoV 229E and PIV4 were not detected in the PSPs. Figure 2 demonstrates the incidence and type of pathogen according to months of the year. Detection of PIV3 increased during the spring and summer and HRV was detected year-round, whereas other viruses were observed with no clear seasonal distribution.

**Table 2:** Number and percentage of each virus detected among 171 PSPs with mid symptoms and no symptoms

All viruses	Positive, no. (%)		Mid Symptoms, no. (%)		Radiographic finding, no. (%)		No Symptoms, no. (%)	
	204	(12.52%)	66	(4.05%)	53	(3.25%)	137	(8.41%)
IV	10	(5.20%)	7	(3.43%)	4	(1.96%)	3	(1.47%)
RSV	19	(9.90%)	16	(7.84%)	6	(2.94%)	3	(1.47%)
HRV/EV	81	(42.19%)	11	(5.39%)	14	(6.86%)	70	(34.31%)
HMPV	5	(2.60%)	4	(1.96%)	1	(0.49%)	1	(0.49%)
ADV	7	(3.65%)	4	(1.96%)	4	(1.96%)	3	(1.47%)
HBoV	8	(4.17%)	4	(1.96%)	3	(1.47%)	4	(1.96%)
PIV1	4	(2.08%)	–	ND^a^	–	ND^a^	4	(1.96%)
PIV2	1	(0.52%)	–	ND^a^	–	ND^a^	1	(0.49%)
PIV3	47	(24.48%)	18	(8.82%)	16	(7.84%)	29	(14.22%)
PIV4	–	ND^a^	–	ND^a^	–	ND^a^	–	ND^a^
Cov OC 43	13	(6.77%)	2	(0.98%)	3	(1.47%)	11	(5.39%)
Cov NL 63	1	(0.52%)	–	ND^a^	–	ND^a^	–	ND^a^
Cov 229E	–	ND	–	ND^a^	–	ND^a^	–	ND^a^
Cov HKU1	8	(4.17%)	–	ND^a^	2	(0.98%)	8	(3.92%)

^a^ND, not detected

### Symptoms and VRI

**Fig. 3:**
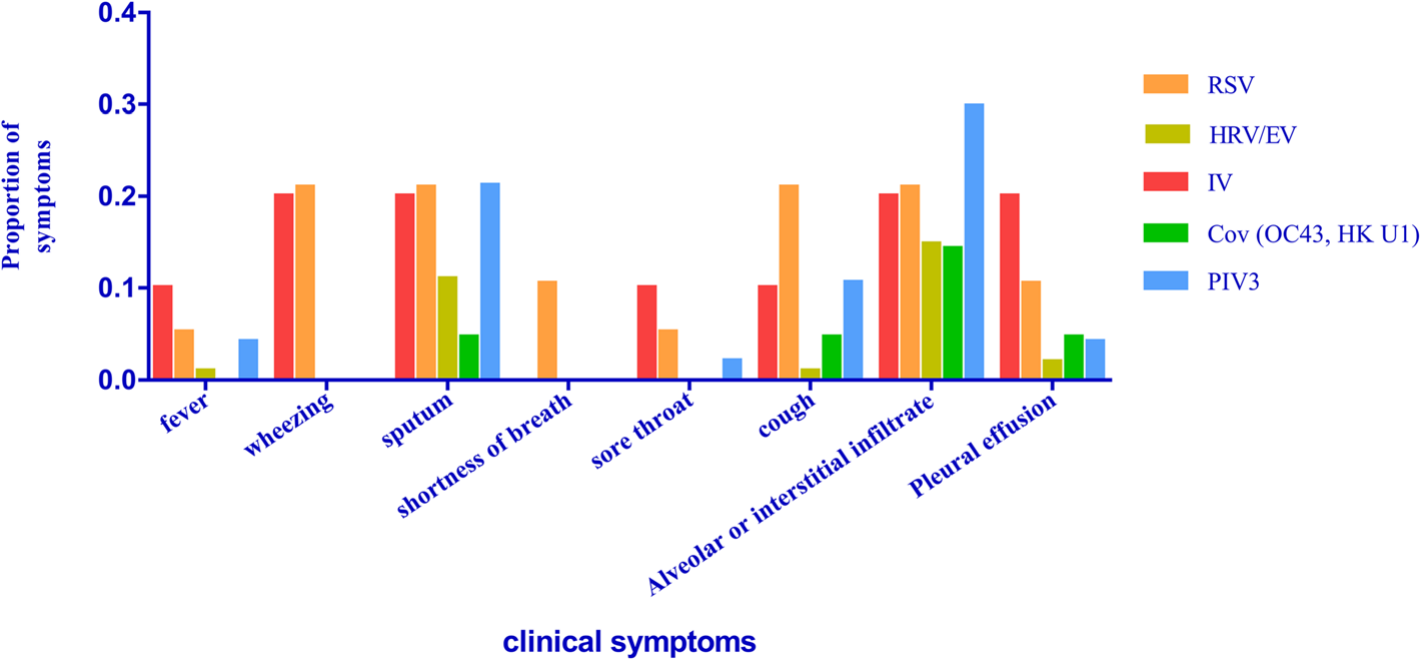
Symptoms and radiographic findings survey versus respiratory infection data collected from PSPs who were detected positive for respiratory viruses. Proportion of symptoms and radiographic findings reported are shown in respiratory samples that tested positive for HRV/EV, RSV, PIV3, CoV HKU1, CoV OC43 and IV

In general, patients reported symptoms more frequently in the survey form when their respiratory samples tested positive than when their samples tested negative. The following viruses were further investigated to explore the association between symptoms and viral infections: HRV/EV(*n* = 81), RSV (*n* = 19), PIV3 (*n* = 47), IV (*n* = 10), and CoV (OC43, HKU1, *n* = 21). These viruses were detected in both asymptomatic patients and patients with mild symptoms (Table 2). Other viruses were not analyzed because of small numbers of positivity. Overall, CoV and HRV/EV were associated with lower proportion of symptoms and PIV3, IV and RSV were associated with higher proportion of symptoms (Fig. 3). RSV-positive surgical patients had more respiratory symptoms than other viruses had, though RSV was also identified in 3 patients without symptom. The proportion of symptoms presenting in the PSPs were lower in samples that tested positive for PIV3 than for IV, probably because of the inclusion of some asymptomatic patients with 29 of PIV3 infection. However, alveolar or interstitial infiltration was most frequently observed in PIV 3-positive patients. The proportion of symptoms was lower for CoV infection than other viruses due to the presence of asymptomatic patients with 11 of CoV infection. A lower proportion of symptoms but higher rate of radiographic findings (alveolar or interstitial infiltration and pleural effusion) were found in HRV/EV-positive patients, though 70 of HRV/EV infections were identified in asymptomatic patients.

### Quantitative respiratory virus load

**Fig. 4:**
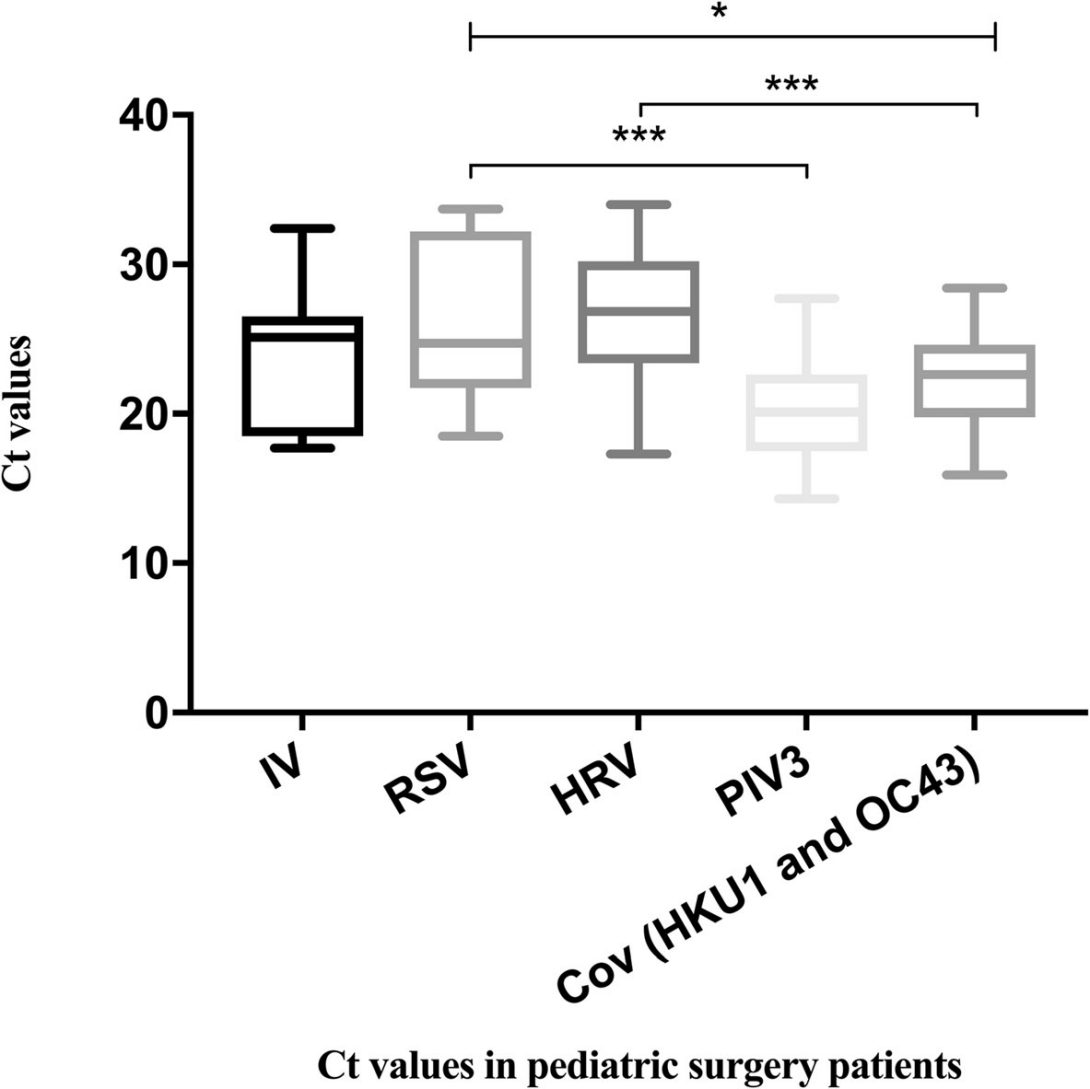
Box and whisker plots form qRT-PCR Ct values among PSPs (age < 18 years) positive for RSV, PIV3, HRV/EV, IV and CoV (HKU1 and OC43). The center of each box plot represents the median, with the box denoting the IQR, and the upper and lower whiskers showing the level 1.5 times the IQR above and below the 75th and 25th percentile, respectively. *P*-values were calculated with the Student-Newman-Keuls test. *p < 0.05, **p < 0.01, ***p < 0.001

To explore a potential association between severity of illness and the virus load, as reflected by the Ct values, Ct values were compared among asymptomatic patients and patients with mild symptoms for the viruses detected in PSPs with a prevalence of > 5%. These viruses included HRV/EV(*n* = 81), RSV (*n* = 19), PIV3 (*n* = 47), IV (*n* = 10), and CoV (OC43, HKU1, n = 21). Figure 4 showed that median Ct values for PIV3- and CoV (HKU1, OC43)-positive PSPs were 20.7 and 22.5, respectively, suggesting a significantly higher viral load than that for RSV (26.1), HRV/EV (27.2), or IV (24.2) (ANOVA, *p* < 0.05). Median Ct values were not significantly different among RVS, HRV/EV and IV (*Mann-Whitney U test*, p < 0.05). PIV3 infection with a higher viral load was frequently found in PSPs presenting with mild symptoms, progressing to pneumonia with radiographic evidence after surgery. In the case of CoV (HKU1, OC43)-positive PSPs, the Ct values were significantly lower than for RSV and IV in patients with severe clinical symptoms supported by radiographic evidence (*Mann-Whitney U test*, *p* < 0.05, Fig. 4). CoV (OC43, HKU1) infections with a higher viral load were also observed in asymptomatic PSPs progressing to alveolar or interstitial infiltration.

## Discussion

VIR has the potential to result in serious respiratory disease in postoperative recovery. In previous studies, the significance of VIR in postoperative recovery patients was questioned, as previous studies reported low positive PCR results for common respiratory viruses in 4.2 and 1.7% of pediatric cardiac surgical patients [15, 16]. These reported studies were limited to RSV, HRV/EV, and IV, possibly missing the other majority of respiratory viruses. In addition, these studies lacked quantitative analysis of viral pathogens in the PSPs. Though the frequency of symptomatic VRI was low, presence of VRI significantly prolonged postoperative recovery in children following surgery, as evidenced by longer LOS periods in hospitalization. In the present study, we aimed to investigate the infection rates and outcomes of both asymptomatic and symptomatic PSPs leading to VRI after surgery using qRT-PCR. As samples were taken not only from patients with symptoms of infection, but also from asymptomatic patients in this study, we were therefore able to explore the impact of viral testing in asymptomatic patients, which could help improve estimates of VRI in PSPs and further study of disease transmission modeling and forecasting.

A total of 171 out of 1629 children (10.50%) were confirmed to have VRI with laboratory results, which is a considerably higher proportion than previous studies have identified. HRV/EV was the most common pathogen detected in 42.19% of PSPs, who had either mild symptoms or were asymptomatic, compared to previously reported 17% of asymptomatic controls and 22% of children with pneumonia enrolled at the same study sites during the same time period [19]. This might be owing to the high sensitivity of the proposed qRT-PCR assays identifying more viral pathogens. In this study, we also observed that HRV/EV were associated with prolonged shedding and the most commonly found coinfections were PIV3 and HRV/EV. These results are consistent with previous findings that HRV/EV co-infection or different HRV species co-infection were associated with acute respiratory illness hospitalization and that prolonged viral shedding over the course of 30 days was more commonly associated with respiratory viruses [9, 20–23]. Because shedding of HRV can occur more than two weeks after infection [19], it could be interpreted that HRV was the most commonly found co-infection pathogen in this study.

RSV and IV infections with higher Ct values and high proportion of symptoms were detected in 9.90 and 5.20% of the PSPs, respectively. RSV was the main pathogen detected in children with pneumonia younger than two years of age. RSV and IV caused more severe clinical symptoms, potentially causing patient transfers to the department of respiratory infection or surgery delays in the preoperative period, suggesting lower detection of RSV and IV than other viruses in this study.

In our study, PIV3 infection with a higher viral load was frequently found in PSPs with mild symptoms, and caused pneumonia with radiographic evidence after surgery. These results were consistent with previous reports that PIV3 was frequently detected in patients with bronchiolitis and pneumonia less than one year of age and was the second pathogen to cause VRIs in neonates and young infants. PIV3 infections were most common in the spring and summer in this study, which is similar to previous report [24]. Additionally, PIV3 infections were also found in 29 patients with no symptoms, this result is different from a previous study in which many viruses (ADV, IV, RSV, HMPV, COV and HRV/EV) but no PIV was identified in hospitalized children without symptoms of respiratory viral illness [11]. Our findings suggested that PIV3 is a new risk factor for VRI in PSPs. The PIV3-infected PSPs with mild symptoms or without obviously clinical respiratory symptoms should strictly adhere to the VRI isolation procedures when undergoing elective surgery. In order to prevent and control VRI, further study of PIV3 infections in the perioperative period is needed.

In a previous study, CoVOC43 was the most commonly detected coronavirus [25], followed by CoV (NL63 HKU1), with similarly moderate detection frequencies. CoV 229E was detected with a comparatively lower frequency [26, 27]. Similar to previous study, CoV OC43 (*n* = 13, 6.77%) and CoV HKU1 (*n* = 8, 4.17%) infections with relatively lower Ct values and less serious illnesses or radiographic findings, were recognized. However, CoV (OC43, HKU1) infections with a higher viral load were also observed in asymptomatic PSPs progressing to alveolar or interstitial infiltration. CoV 229E was not discovered in PSPs in this study.

This observational study has several limitations: 1) The small subgroup numbers may lead to differences in detection rates, making it worthwhile to consider a multicenter study with greater numbers of surgical cases to further examine the impact of VRI on postoperative outcomes; 2) The possible bacterial pathogens in the PSP were not considered; 3) no information was available about the vaccination and immunological status of eligible patients.

In conclusion, our findings indicate that VRI is associated with prolonged postoperative recovery in children following surgery. In addition, PIV3 is a new risk factor for VRI in PSPs. Employing a more sensitive and quantitative method, such as qRT-PCR, should be considered for preoperative testing of respiratory viruses. Further prospective studies are required to define the risk stratification and to guide optimal surgical timing.

## Data Availability

The datasets used and/or analyzed during the current study are available from the corresponding author on reasonable request.

## Abbreviations

ADV: Human adenovirus
CDC: Centers for Disease Control and Prevention
CoV: Coronavirus
Ct: Threshold cycle
HBoV: Human bocavirus
HMPV: Human metapneumovirus
HRV: Human rhinovirus
HRV/EV: Human rhino/enterovirus
IQR: Interquartile range
IV: Influenza virus
LOS: Length of stay
PIV3: Parainfluenza virus 3
PSPs: Pediatric surgery patients
qRT-PCR: Quantitative real-time reverse transcriptase polymerase chain reaction
RSV: Respiratory syncytial virus
VRI: Viral respiratory infection
